# Incidence of Panic Disorder Diagnoses After Celebrity Disclosures of Panic Disorder in South Korea

**DOI:** 10.1001/jamanetworkopen.2024.20934

**Published:** 2024-07-10

**Authors:** Ga Eun Kim, Min-Woo Jo, Young Eun Kim, Seok-Jun Yoon, Yong-Wook Shin

**Affiliations:** 1 Department of Psychiatry, Ewha Womans University Mokdong Hospital, Ewha Womans University College of Medicine, Seoul, Korea; 2 Department of Preventive Medicine, University of Ulsan College of Medicine, Seoul, Korea; 3 Department of Big Data Strategy, National Health Insurance Service, Wonju, Korea; 4 Department of Preventive Medicine, Korea University College of Medicine, Seoul, Korea; 5 Department of Psychiatry, Asan Medical Centre, University of Ulsan College of Medicine, Seoul, Korea

## Abstract

**Importance:**

The persistent stigma associated with mental health conditions is a major challenge worldwide. Celebrities may improve this by openly discussing their own mental health issues, potentially influencing public attitudes and encouraging individuals to seek treatment for these conditions.

**Objective:**

To evaluate the impact of celebrity mental health disclosures on the incidence and prevalence of panic disorder diagnosis in South Korea.

**Design, Setting, and Participants:**

This cohort study included the entire South Korean population from January 2004 to December 2021, as reflected in the National Health Insurance Service data. Analysis was conducted from May 2022 through January 2024.

**Exposure:**

Time periods analyzed included the timeframe before (from January 2004 to December 2010) and after the public disclosures of panic disorder by 3 high-profile Korean celebrities between December 2010 and January 2012 (from January 2011 to December 2021).

**Main Outcomes and Measures:**

Monthly incidence and prevalence of panic disorder, defined by the presence of a clinical diagnosis of the condition. Trends were assessed using interrupted time series analysis with autoregressive integrated moving average models. To assess public interest in panic disorder, trends in search data were analyzed, examining the association between the timing of increased searches and changes in the incidence and prevalence of panic disorder. Data on obsessive-compulsive disorder (OCD) were included as a control.

**Results:**

The study covered the entire population of South Korea, including 48 559 946 individuals in January 2004 and 52 593 886 individuals in December 2021. Before 2011, the mean (SD) annual prevalence of panic disorder was stable at 560 (140) persons per 100 000 persons per year. The celebrity disclosure in December 2010 was associated with higher monthly incidence rates of panic disorder, as measured by insurance claims data, changes that were observed in both the level (5.8 persons; 95% CI, 2.2-9.5 persons) and slope (0.78 persons per month; 95% CI, 0.19-1.40 persons per month) per 100 000 persons. By 2021, the observed annual prevalence per 100 000 persons reached 7530 persons, an increase of 775.6% compared with the 860 persons (95% CI, 330-1400 persons) estimated if the disclosures had not occurred. Internet searches anticipated changes in monthly prevalence with a lag of 2 or 3 months (F = 4.26, *P* = .02 and F = 3.11, *P* = .03, respectively). The celebrity disclosures had no significant association with the incidence or prevalence of OCD.

**Conclusions and Relevance:**

In this observational cohort study, celebrity disclosure of mental health conditions was associated with a sustained reduction in stigma, as reflected in increased help-seeking behavior for the condition over more than a decade. This underscores the influential role celebrities can play in shaping public health perceptions and behaviors, offering valuable insights for the development of future mental health policies and public awareness campaigns.

## Key Points

**Question:**

How do celebrity disclosures of mental health conditions correlate with public attitudes and help-seeking behavior for these conditions in a population?

**Findings:**

In this cohort study, there was a significant and sustained increase in both the incidence and prevalence of panic disorder diagnoses in South Korea following the public disclosure of panic disorder diagnoses by 3 high-profile Korean celebrities between December 2010 and January 2012, as evidenced by insurance claims data. Internet searches for the condition surged during this period.

**Meaning:**

These results suggest that celebrity disclosures are associated with immediate and lasting reductions in stigma around mental health conditions, as reflected in increased help-seeking behavior, and may positively influence public attitudes toward mental health conditions for more than a decade.

## Teaser

This cohort study of national claims data in South Korea examines the association of panic disorder diagnoses and related internet search traffic with 3 public disclosures of panic disorder diagnosis by celebrities.

## Introduction

The stigma related to mental health conditions, which has a significant impact on individuals with the conditions, persists worldwide. The effects of this stigma are far-reaching, ranging from social exclusion to reduced life expectancy.^1^ The stigmatization of individuals with mental health conditions results in a fear of feeling inferior and morally culpable, which can prevent them from seeking help.^2^ Such negative stereotypes have been reinforced by the media, which often portrays those with mental health conditions as potentially dangerous and morally responsible for their conditions.^3^ Celebrities and the media’s portrayal of them have also contributed to the negative impact on people’s mental health and perceptions of mental health conditions.^4^ For instance, people may imitate the maladaptive behaviors of celebrities they admire, such as substance abuse^5^ or even committing suicide.^4^

However, health professionals involved in patient advocacy have argued that celebrities “can be enormously helpful to ordinary people with the same condition.”^6^ By disclosing personal stories of people with lived experience (PWLE), celebrities can particularly challenge stigma; people recognize that anyone, including successful, high-profile individuals, can experience mental health problems. Celebrities have disclosed their mental health conditions in the media.^7^ In 1995, Diana, Princess of Wales, revealed her struggle with bulimia in the media. Following her disclosure, there appeared to be an increase in the number of individuals seeking treatment for bulimia in the UK, which was called the “Diana effect,” although the relationship between the disclosure and the seeking-help behavior has not been established.^8^ Demi Lovato and Selena Gomez, both influential figures in the entertainment industry, have also spoken openly about being diagnosed with bipolar disorder and depression.^9^ However, despite the large number of these reports and theories suggesting that celebrities can normalize psychiatric diagnoses and promote treatment-seeking behaviors,^10,11^ there is little evidence on the long-term impact of such disclosures on public attitudes and behaviors toward mental health conditions.^1^

In December 2010, a popular South Korean male actor, Tae-hyun Cha, disclosed that he had been diagnosed with panic disorder.^12^ Before the disclosure, public interest in mental health conditions in South Korea was low.^13^ In October 2011 and January 2012, following the previous disclosure, a popular singer, Jang-hoon Kim, and comedian, Kyung-kyu Lee, also went public with their struggles with panic disorder, attracting further national media attention.^14^ Below is an extract from a newspaper article published on December 3, 2010, one of a series of 10 or more articles published that month about the initial disclosure:

“Actor Cha Tae-hyun, riding a wave of renewed popularity...revealed that he struggled with panic disorder before his marriage. ...However, what exactly is panic disorder, the condition Cha Tae-hyun admitted to battling after a series of his box office failures? Panic disorder is characterized by spontaneous bouts of intense fear or discomfort, known as panic attacks, which occur without any specific trigger. Symptoms may include chest tightness or severe headaches. Even if there is no immediate danger, a person may suddenly experience a racing heart, throbbing headache, and profuse sweating in the hands and feet, all signs of a panic attack. If such episodes occur two or three times a month, a diagnosis of panic disorder may be made…”^12^

South Korea’s health care system is characterized by a single, mandatory payer system, with virtually the entire population enrolled in the National Health Insurance Service (NHIS).^15^ This near-universal coverage ensures that changes in health-seeking behavior are directly and promptly reflected in the NHIS data. We analyzed NHIS data covering the entire South Korean population to explore potential shifts in the incidence and prevalence of panic disorder before and after celebrity disclosures from 2004 to 2021. To ensure the specificity of our results, we also examined the incidence and prevalence of OCD, which received minimal media coverage during the study period.

## Methods

### Study Design and Population

To determine the incidence and prevalence of panic disorders in South Korea, we conducted a retrospective observational cohort study from 2004 to 2021 using claims data from the NHIS. As the NHIS is the payer for both insured individuals and Medicaid beneficiaries, this database encompasses claims-level records for the entire population of South Korea.^15^ The database comprises demographic information and health care utilization data from both inpatient and outpatient settings. This includes diagnostic codes, drug prescriptions, medical tests, prescribed procedures, and health care expenditures authorized by the NHIS health care system.^15^ Data from the NHIS have been validated in previous studies including both medical and psychiatric diagnoses.^16,17,18^ The institutional review board of Korea University approved this study. Informed consent was waived by the board because the claims data used were deidentified. This study adheres to the Strengthening the Reporting of Observational Studies in Epidemiology (STROBE) reporting guideline.

### Definition of Panic Disorder and OCD

Diagnostic information in the NHIS health care system is coded according to the *International Statistical Classification of Diseases and Related Health Problems, Tenth Revision (ICD-10)*. Panic disorder was classified using the *ICD-10* diagnostic code F41.0. We chose individuals diagnosed with OCD as a control group for panic disorder because they share epidemiological and clinical characteristics, including relatively comparable prevalence,^19^ a similar age of onset,^20^ and a tendency toward outpatient treatment; however, these 2 diagnoses have distinct clinical manifestations with minimal overlap.^21^ OCD was designated by the *ICD-10* diagnostic codes F42, F42.0, F42.1, F42.2, F42.8, and F42.9. In the NHIS, medical service professionals register diagnoses in an order that indicates the importance of each diagnosis to the patient’s medical visit. To identify the occurrence of panic disorder and OCD, we evaluated up to 4 levels of entered diagnosis (primary, secondary, tertiary, and quaternary).

### Incidence and Prevalence of Panic Disorder and OCD

Monthly prevalence was calculated by dividing the total number of individuals diagnosed with either panic disorder or OCD in each month by the total registered population for the same month. Monthly incidence was calculated by dividing the number of individuals diagnosed in each month who had not been diagnosed in the previous 2 years by the total registered population.

### Internet Search Trends Data on Panic Disorder and OCD

To evaluate shifts in public interest regarding panic disorder before and after celebrity disclosures, we used Google Trends. This tool displays the relative search volume and query share for a specified search term, tailored to a particular location and time frame. The data are normalized based on the term’s peak share over the selected period. We obtained the trend for the search terms *panic disorder* and *obsessive-compulsive disorder* in Korean from January 2004 to December 2021. The search location was South Korea, encompassing all query categories. The data were accessed and downloaded on August 12, 2022.

### Statistical Analysis

We used interrupted time series analysis combined with autoregressive integrated moving average (ARIMA) modeling to assess changes in levels and trends of monthly incidence and prevalence of panic disorder following celebrity disclosures that occurred in December 2010, October 2011, and January 2012. The ARIMA model analyzed the intervention effect of these celebrity disclosures, considering seasonal trends and autocorrelation within the time series data. The optimal ARIMA model was determined by minimizing the information criteria (Akaike information criterion and bayesian information criterion) using the forecast package in R. Model fit was assessed by examining residual plots and testing for the presence of autocorrelation using the Ljung box test to determine whether residuals were consistent with white noise.

Using the optimal ARIMA parameters, we estimated the monthly incidence and prevalence of panic disorder that might have occurred in the absence of celebrity disclosures. We compared these with the observed monthly incidence and prevalence of panic disorder. As a sensitivity analysis, we performed a change-point analysis using the changepoint package in R. This aimed to determine any noticeable change in the time series pattern from a data perspective and identify the optimal time of change; the at most one change (AMOC) method was used to detect the single change point, considering both mean and variance changes.^22^

To determine whether the incidence or prevalence of panic disorder was influenced by public interest in the diagnosis of the celebrities, we examined possible associations between search trends data and the monthly incidence or prevalence of panic disorder. A Granger causality test was conducted to examine time-directional causality from search trends data to changes in monthly incidence or prevalence. Since the stationarity of the variable failed the Augmented Dickey-Fuller (ADF) test using the R package tseries, the first-order difference of the variables was tested and used. The Granger causality test was performed using the R package lmtest. The magnitude of the first-order difference in the monthly prevalence of panic disorder and Google search volume before and after the first celebrity disclosure were compared using Student *t* test. All statistical tests used in the analysis were 2-tailed, with an a priori significance level set at .05. All statistical analyses were performed using the R package version 4.3.2 (R Project for Statistical Computing).

## Results

Total cohort size ranged from 48 559 946 individuals in January 2004 (mean [SD] age, 34.7 [20.1] years; 24 160 508 women [49.8%]) to 52 593 886 individuals in December 2021 (mean [SD] age, 43.3 [21.5] years; 26 335 661 women [50.1%]).

### Celebrity Panic Disorder Disclosures and Search Data

**Figure 1.**
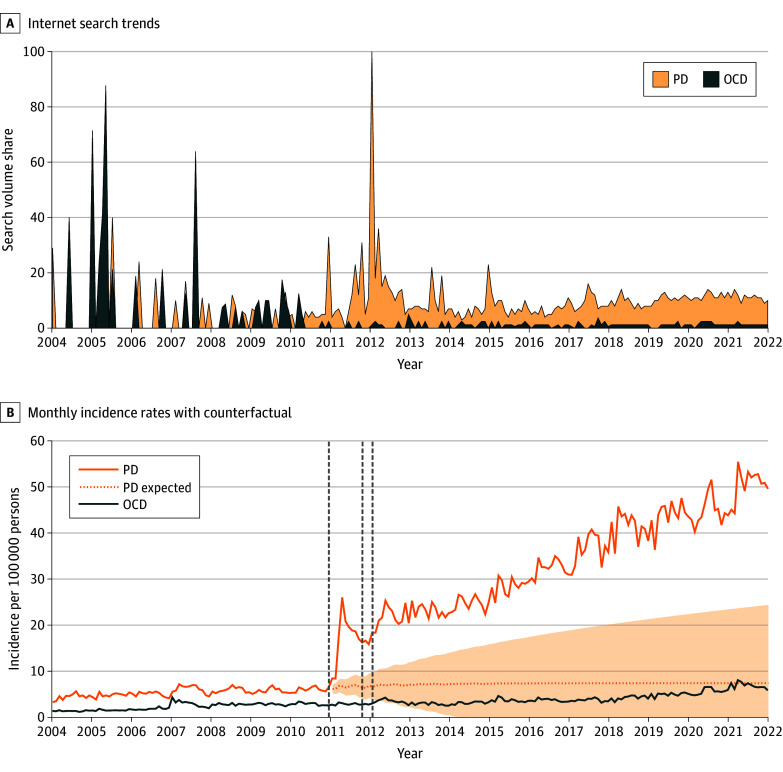
Internet Search Trends and Monthly Incidence for Panic Disorder (PD) and Obsessive-Compulsive Disorder (OCD) Before and After Celebrity Disclosures of Panic Disorder Expected PD shows the counterfactual trend without the celebrity exposure based on the autoregressive integrated moving average model, with shading indicating 95% CIs. Dates of the celebrities’ disclosures are marked with gray dotted vertical lines. These dates—December 2010, October 2011, and January 2012—correspond to when a film actor, a singer, and a comedian made their panic disorder diagnosis public through the mainstream media.

Within the timeframe corresponding to the celebrities’ disclosures between December 2010 and January 2012, the spikes in Google Trends data for *panic disorder* coincided with the timing of the celebrities’ public disclosures (Figure 1A). Using the peak detection algorithm in the R package cardidates, 3 peaks were identified, December 2010, October 2011, and January 2012.

### Incidence of Panic Disorder Following Celebrity Disclosures

The mean (SD) monthly incidence of panic disorder diagnoses remained stable from January 2004 to December 2010 at 5.4 (0.9) persons per 100 000 persons. After the first celebrity disclosure in December 2010, the incidence increased from 6.5 persons per 100 000 persons in December 2010 to 8.4 persons per 100 000 persons in January 2011 (an increase of 29.2%), 8.4 persons per 100 000 persons in February 2011 (29.2%), 18.0 persons per 100 000 persons in March (176.9%), and 26.0 persons per 100 000 persons in April 2011 (300%). The trend of increasing incidence rates of panic disorder continued, reaching an annual rate of 610 persons per 100 000 in 2021. This represents an increase of 838.5% compared with the mean (SD) annual incidence of panic disorder of 65 (8.2) persons per 100 000 recorded between 2004 and 2010 (Figure 1B; eTable 1 in Supplement 1).

Figure 1B shows the fit of the ARIMA model and the forecasted monthly incidence of panic disorder in the absence of celebrity disclosure. The optimal ARIMA model for the monthly incidence of panic disorder, ARIMA (1,0,0) × (0,0,1)_12_, showed a significant effect of celebrity disclosure. The first celebrity disclosure was associated with a higher level (5.8 persons per 100 000 persons; 95% CI, 2.2-9.5 persons; *P* = .002) and steeper slope (0.78 persons per 100 000 persons per month; 95% CI, 0.19 to 1.4 persons per month; *P* = .009) in the monthly incidence of panic disorder. The other 2 disclosures showed no significant association. The observed annual incidence of 610 persons per 100 000 persons in 2021 was 577.8% higher than the predicted incidence of 90 persons per 100 000 persons (95% CI, −110 to 290 persons). The change-point analysis identified December 2010 as the most likely time of change in monthly incidence during the study period (eFigure 1 in Supplement 1).

There was a slight fluctuation in the monthly incidence of OCD observed after the first celebrity disclosure, with the monthly incidences of 2.8 persons per 100 000 persons in January, 2.6 persons per 100 000 persons in February, 3.3 persons per 100 000 persons in March, and 3.1 persons per 100 000 persons in April 2011, compared with 2.6 persons per 100 000 persons in December 2010. Nevertheless, in the identified model for OCD, ARIMA (1,0,2) × (0,0,2)_12_, none of the celebrity disclosures were significantly associated with changes in the monthly incidence of OCD (eTable 2 in Supplement 1).

### Prevalence of Panic Disorder Following Celebrity Disclosures

**Figure 2.**
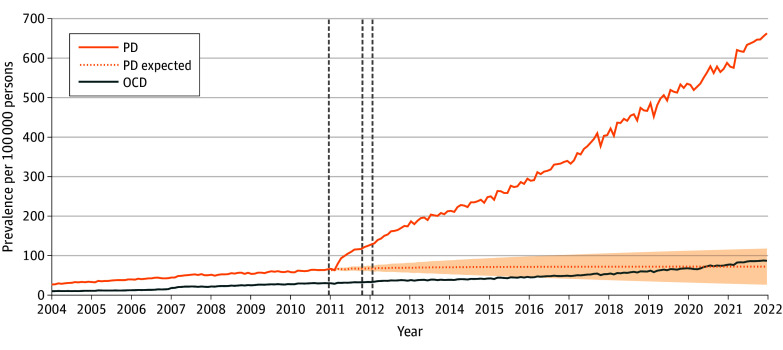
Monthly Prevalence of Panic Disorder (PD) and Obsessive-Compulsive Disorder (OCD) Before and After Celebrity Disclosure of Panic Disorder Expected PD shows the counterfactual trend without the celebrity exposure based on the autoregressive integrated moving average model, with shading indicating 95% CIs. Dates of the celebrities’ disclosures are marked with gray dotted vertical lines. These dates—December 2010, October 2011, and January 2012—correspond to when a film actor, a singer, and a comedian made their panic disorder diagnosis public through the mainstream media.

From 2004 to 2010, the mean (SD) annual prevalence of panic disorder was 560 (140) persons per 100 000 persons. Following the first celebrity disclosure in December 2010, the annual prevalence increased from 740 persons per 100 000 persons in 2010 to 1200 persons per 100 000 persons in 2011 (an increase of 62.2%), 1850 persons per 100 000 persons in 2012 (150%), and 2360 persons per 100 000 persons in 2013 (218.9%); the trend continued to increase, reaching an annual prevalence of 7530 persons per 100 000 persons in 2021 (917.6%) (Figure 2; eTable 3 in Supplement 1)

The optimal ARIMA model for the monthly prevalence of panic disorder was ARIMA (0,1,1) × (0,0,1)_12_ (Figure 2). In the model, the first celebrity disclosure was associated with a change in the slope of the prevalence (5.9 persons per 100 000 persons per month; 95% CI, 4.0 to 7.8 persons per month; *P* < .001), but there was no significant association with the level (−2.9 persons per 100 000 persons; 95% CI, −14 to 8.5 persons; *P* = .62). The second and third disclosures were not associated with significant changes in the level or slope of the prevalence. The observed annual prevalence of 7530 persons per 100 000 persons in 2021 was 775.6% higher than the estimated prevalence of 860 persons per 100 000 persons (95% CI, 330 to 1400 persons). The change-point analysis identified March 2011 as the most likely time for a shift in the monthly prevalence of panic disorder (eFigure 2 in Supplement 1). Celebrity disclosures of panic disorder were not significantly associated with changes in OCD prevalence in the identified model of ARIMA (0,1,1) × (0,0,1)_12_ (eTable 4 in Supplement 1).

### Internet Searches and Prevalence of Panic Disorder

**Figure 3.**
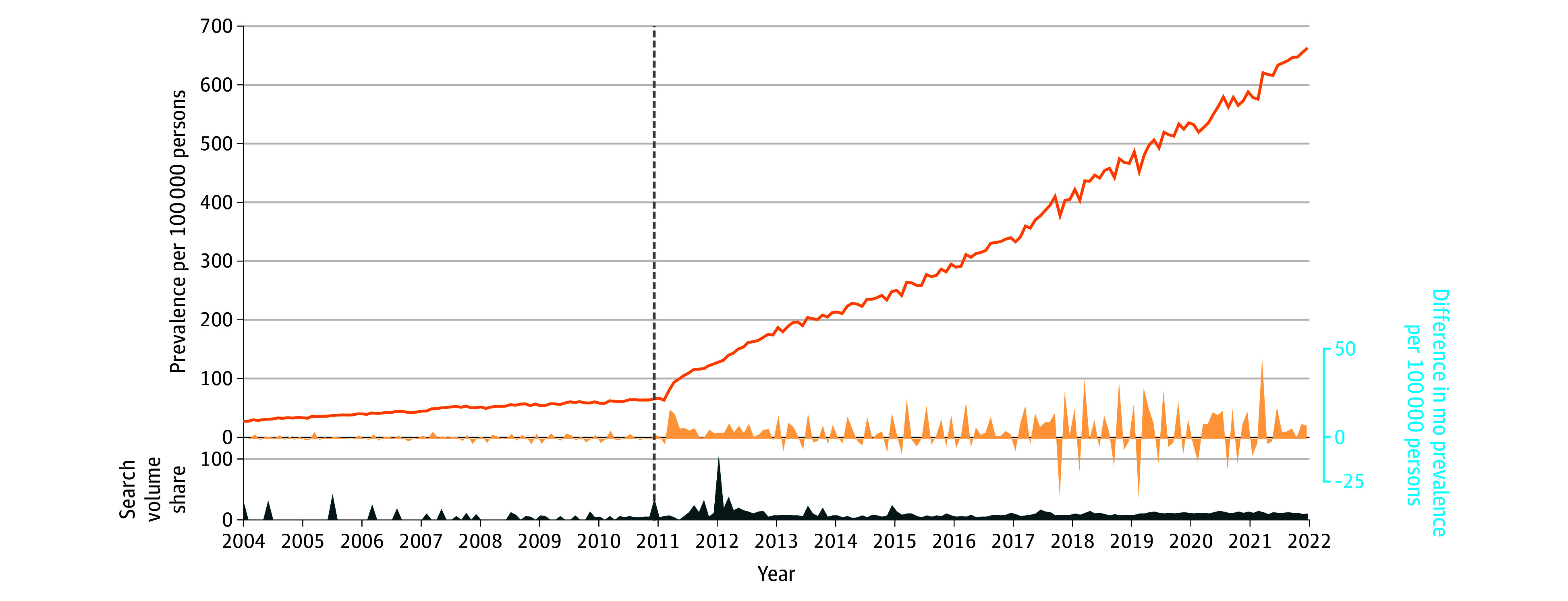
Internet Search Trends and the Overall Prevalence and First-Order Difference in Monthly Prevalence for Panic Disorder The filled orange area indicates the first-order difference for PD; the dotted line represents the date, December 2010, when a film actor made his panic disorder diagnosis public through the media.

Monthly incidence and prevalence of panic disorder were nonstationary (ADF statistic = −2.98, *P* = .17 and ADF statistic = −0.73, *P* = .97 for incidence and prevalence, respectively), whereas the first-order differences of these were stationary (ADF statistic = −8.19, *P* < .01 [the lowest *P *value limit in the R package that performs this test^44^] and ADF statistic = −7.14, *P* < .01 for incidence and prevalence, respectively). Following the first celebrity disclosure in December 2010, there was an increase in search volume (mean [SD] index value, 4.12 [8.06] during the period from 2004 to 2010 vs 10.46 [9.24], from 2011 to 2021; *t* = −5.32, *P* < .001) and in the magnitude of the first-order difference in monthly prevalence (mean [SD], 1.2 [1.0] vs 8.9 [8.2] persons per 100 000 persons; *t* = −10.67, *P* < .001) (Figure 3). The Granger causality test showed that the internet search trends data was a significant indicator for the first-order difference in the monthly prevalence of panic disorder with a lag of 2 (F = 4.26, *P* = .02) or 3 (F = 3.11, *P* = .03) months. The inverse was not significant. No significant Granger causality was found between internet search trends and panic disorder incidence, nor between internet search trends and OCD incidence or prevalence.

## Discussion

Following the 3 celebrity disclosures, there was an increase in help-seeking behavior in South Korea, indicated by an increase in the monthly incidence and prevalence of panic disorder as measured by insurance claims data. This increase persisted for over a decade after the disclosures. This suggests that celebrity disclosures have had a powerful influence in shaping public attitudes and reducing stigma around mental health issues. By speaking openly as PWLE about their own experiences with panic disorder, these high-profile individuals are likely to have encouraged people to recognize symptoms and seek assessment and treatment. The simultaneous increase in online searches, alongside Granger causality analysis, supports the view that the disclosures were associated with increased public interest and awareness of panic disorder.

The annual prevalence of panic disorder in South Korea was initially lower than in Western countries,^23^ similar to other East Asian countries^24,25,26^ where greater stigma has persisted.^27,28,29^ Following the celebrity disclosures, the prevalence in South Korea had surpassed rates in the US, the UK, and other Western countries.^30,31^ Our study’s findings before the celebrity disclosures are in line with those of previous survey studies that reported lower prevalence rates of panic disorder in South Korea compared with Western countries.^23,32^ However, the shift in incidence and prevalence observed in our study suggests that many cases of panic disorder in South Korea may have previously been unrecognized and untreated.^33^ Celebrity disclosures appear to play a role in bridging the treatment gap for individuals with panic disorder. The low prevalence reported in survey studies, even after celebrity disclosures, suggests that fear of disclosure persists. It is possible that increased interest and awareness of panic disorder may have contributed more to increased help-seeking behavior than decreased stigma in the general population.

The incidence and prevalence of panic disorder and OCD did not experience significant changes during the COVID-19 pandemic compared with those observed after celebrity disclosures. Despite reports of increased anxiety symptoms during the pandemic,^34,35,36^ these did not translate to a major rise in mental health service use. Studies, including one using NHIS data,^37^ found minimal changes in mental health service use, in line with the findings of a recent multinational study showing an initial decrease in mental health diagnoses during the pandemic, followed by a gradual recovery.^38^

With their extensive reach and influence, celebrities were seen as capable of reshaping public attitudes and beliefs about mental health conditions.^39^ However, much of the research supporting these observations is anecdotal and lacks methodological rigor; there’s also a lack of evidence on the long-term impact of celebrity disclosures in reducing stigma or improving mental health literacy among the general population.^8,10^ Antistigma campaigns on mental health have reported less optimistic long-term impacts on both stigmatization and health-seeking behavior.^1,40,41,42^ Our study demonstrates the long-term association between celebrity disclosures and increased treatment-seeking behavior for panic disorder. This association may contribute to improvements in mental health literacy and potentially reduce stigma in the general population. In our analysis, celebrities played an enduring role for over a decade by increasing public engagement with mental health issues. Their continued fame and success may reinforce the longer-term influence by showing the public that revealing a psychiatric diagnosis does not damage one’s career. Given their lasting influences, thoughtful employment of celebrity disclosures shows promise as a key element in successful campaigns aimed at combating stigma and enhancing public understanding of mental health conditions. As celebrities increasingly use their platforms to share their personal stories, the evidence suggests a need for more celebrity-led campaigns and messaging designed specifically to promote mental health help-seeking, education, and acceptance. It is important to approach the interpretation of our findings with care. Criticism that celebrity disclosures unnecessarily increase rates of psychiatric diagnoses can perpetuate stigma and detract from the value of their advocacy. This arbitrary and politicized interpretation may harm efforts to promote mental health awareness.

### Limitations

This study using a claims dataset has several limitations. These include the risk of misdiagnosis by health care professionals and a potential underestimation of prevalence due to barriers to accessing health care. Although claims data cannot fully capture those who avoid seeking care, this limitation ironically allows us to examine health-seeking behavior with mental health stigma. There is also a potential for overdiagnosis, as we included up to 4 levels of clinician diagnosis in the claim. However, our main finding—the shift in incidence and prevalence following celebrity disclosures—is robust to these misclassifications of underrepresentation or overrepresentation bias, provided that such biases do not change significantly between the predisclosure and postdisclosure periods. There remains a possibility that both clinicians and patients may be more inclined to diagnose panic disorder than other diagnoses, influenced by the reduced stigma specific to panic disorder, potentially leading to overdiagnosis in cases with nonspecific anxiety symptoms. The absence of similar trends in OCD rules out potential confounding factors such as economic fluctuations or changes in health policy. This strongly suggests that the change in panic disorder prevalence was due to these disclosures. Similar to our results, a study using both survey and registry data in South Korea reported that the annual prevalence of panic disorder showed a much steeper increase in registry data than other psychiatric diagnoses from 2006 to 2016.^18^ However, our findings should be interpreted with caution, given the potential influence of unexamined confounders and possible inconsistencies in data sources. A point of intrigue is why only the first of the 3 disclosures registered a significant effect in the ARIMA model. One possible explanation is the close temporal proximity of the events, which may have led to cumulative effects that are statistically indistinguishable.^43^

## Conclusions

Using national claims data, this study explored the association of celebrity mental health disclosures in the media with public mental health-related behaviors. Our findings suggest the continued potential for celebrities to influence public attitudes, reduce stigma, and promote positive health behaviors concerning mental health conditions. Further research can build on these findings to better understand the nuanced impact that celebrities can have on critical mental health issues that affect all members of the population beyond their typical demographic of influence.

## Notes

Supplement 1. **eTable 1.** Annual Incidence of PD **eTable 2.** Annual Incidence of OCD **eTable 3.** Annual Prevalence of PD **eTable 4.** Annual Prevalence of OCD **eFigure 1.** Change-Point Analysis of the Incidence of PD **eFigure 2.** Change-Point Analysis of the Prevalence of PD

Supplement 2.
Data Sharing Statement

## References

[1] Gronholm PC, Thornicroft G. Impact of celebrity disclosure on mental health-related stigma. Epidemiol Psychiatr Sci. 2022;31:e62. doi:10.1017/S2045796022000488 36039976 PMC9483822

[2] Clement S, Schauman O, Graham T, . What is the impact of mental health-related stigma on help-seeking? A systematic review of quantitative and qualitative studies. Psychol Med. 2015;45(1):11-27. doi:10.1017/S0033291714000129 24569086

[3] Coverdale J, Nairn R, Claasen D. Depictions of mental illness in print media: a prospective national sample. Aust N Z J Psychiatry. 2002;36(5):697-700. doi:10.1046/j.1440-1614.2002.00998.x 12225457

[4] Dillman Carpentier FR, Parrott MS. Young adults’ information seeking following celebrity suicide: considering involvement with the celebrity and emotional distress in health communication strategies. Health Commun. 2016;31(11):1334-1344. doi:10.1080/10410236.2015.1056329 26984641

[5] Zsila Á, Orosz G, McCutcheon LE, Demetrovics Z. A lethal imitation game? Exploring links among psychoactive substance use, self-harming behaviors and celebrity worship. Addict Behav Rep. 2020;12:100319. doi:10.1016/j.abrep.2020.100319 33364327 PMC7752730

[6] Cayton H. Celebrity illness. Lancet. 1995;346(8976):705. doi:10.1016/S0140-6736(95)92315-2 7658844

[7] Thelandersson F. Celebrity Mental Health: Intimacy, Ordinariness, and Repeated Self-Transformation. In: 21st Century Media and Female Mental Health: Profitable Vulnerability and Sad Girl Culture. Palgrave Macmillan; 2022: 103-155. doi:10.1007/978-3-031-16756-0_4

[8] Currin L, Schmidt U, Treasure J, Jick H. Time trends in eating disorder incidence. Br J Psychiatry. 2005;186:132-135. doi:10.1192/bjp.186.2.132 15684236

[9] Franssen G. The celebritization of self-care: The celebrity health narrative of Demi Lovato and the sickscape of mental illness. Eur J Cult Stud. 2020;23(1):89-111. doi:10.1177/1367549419861636

[10] Calhoun AJ, Gold JA. “I feel like I know them”: the positive effect of celebrity self-disclosure of mental illness. Acad Psychiatry. 2020;44(2):237-241. doi:10.1007/s40596-020-01200-5 32100256

[11] Nelson A. Ups and downs: social media advocacy of bipolar disorder on World Mental Health Day. Front Commun (Lausanne). 2019;4:24. doi:10.3389/fcomm.2019.00024

[12] Han HJ. What was the ‘panic disorder’ that plagued Cha? Healthchosun. December 3, 2010. Accessed May 26, 2023. https://m.health.chosun.com/svc/news_view.html?contid=2010120300347&

[13] Baik J, Lee H-Y, Nam H-E. Research trend analysis of health and mental health literacy in Korea: 2007-2017. Korean J Health Service Manage. 2018;12(3):95-106. doi:10.12811/kshsm.2018.12.3.095

[14] Shin Hi. Shadow behind the light: celebrities fighting panic disorder. The Korea Herald. January 9, 2012. Accessed May 13, 2023. https://www.koreaherald.com/view.php?ud=20120109000872

[15] Kyoung DS, Kim HS. Understanding and utilizing claim data from the Korean National Health Insurance Service (NHIS) and Health Insurance Review & Assessment (HIRA) database for research. J Lipid Atheroscler. 2022;11(2):103-110. doi:10.12997/jla.2022.11.2.103 35656154 PMC9133780

[16] Lee JY, Ju HJ, Han JH, . Autoimmune, inflammatory, atopic, thyroid, and psychiatric outcomes of offspring born to mothers with alopecia areata. JAMA Dermatol. 2023;159(7):711-719. doi:10.1001/jamadermatol.2023.1261 37223925 PMC10209830

[17] Cha W, Yun I, Nam CM, Nam JY, Park EC. Evaluation of assisted reproductive technology health insurance coverage for multiple pregnancies and births in Korea. JAMA Netw Open. 2023;6(6):e2316696. doi:10.1001/jamanetworkopen.2023.16696 37279002 PMC10245192

[18] Jo M, Rim SJ, Lee MG, Park S. Illuminating the treatment gap of mental disorders: a comparison of community survey-based and national registry-based prevalence rates in Korea. J Psychiatr Res. 2020;130:381-386. doi:10.1016/j.jpsychires.2020.07.047 32882580

[19] Martin P. The epidemiology of anxiety disorders: a review. Dialogues Clin Neurosci. 2003;5(3):281-298. doi:10.31887/DCNS.2003.5.3/pmartin 22034470 PMC3181629

[20] Lijster JM, Dierckx B, Utens EM, . The age of onset of anxiety disorders. Can J Psychiatry. 2017;62(4):237-246. doi:10.1177/0706743716640757 27310233 PMC5407545

[21] Kennedy BL, Morris RL, Pedley LL, Schwab JJ. The ability of the Symptom Checklist SCL-90 to differentiate various anxiety and depressive disorders. Psychiatr Q. 2001;72(3):277-288. doi:10.1023/A:1010357216925 11467161

[22] Eckley IA, Fearnhead P, Killick R. Analysis of changepoint models. In: Barber D, Cemgil AT, Chiappa S, eds. Bayesian time series models. Cambridge University Press; 2011:205-224. doi:10.1017/CBO9780511984679.011

[23] Cho MJ, Seong SJ, Park JE, . Prevalence and correlates of DSM-IV Mental Disorders in South Korean adults: the Korean Epidemiologic Catchment Area Study 2011. Psychiatry Investig. 2015;12(2):164-170. doi:10.4306/pi.2015.12.2.164 25866515 PMC4390585

[24] Shen YC, Zhang MY, Huang YQ, . Twelve-month prevalence, severity, and unmet need for treatment of mental disorders in metropolitan China. Psychol Med. 2006;36(2):257-267. doi:10.1017/S0033291705006367 16332281

[25] Ishikawa H, Tachimori H, Takeshima T, . Prevalence, treatment, and the correlates of common mental disorders in the mid 2010's in Japan: the results of the world mental health Japan 2nd survey. J Affect Disord. 2018;241:554-562. doi:10.1016/j.jad.2018.08.050 30153639

[26] Horwath E, Cohen RS, Weissman MM. Epidemiology of Depressive and Anxiety Disorders. Textbook in Psychiatric Epidemiology. Wiley Online Library; 2002:389-426.

[27] Krendl AC, Pescosolido BA. Countries and cultural differences in the stigma of mental illness: the East–West Divide. J Cross Cult Psychol. 2020;51(2):149-167. doi:10.1177/0022022119901297

[28] Ran MS, Hall BJ, Su TT, . Stigma of mental illness and cultural factors in Pacific Rim region: a systematic review. BMC Psychiatry. 2021;21(1):8. doi:10.1186/s12888-020-02991-5 33413195 PMC7789475

[29] Dubreucq J, Plasse J, Franck N. Self-stigma in serious mental illness: a systematic review of frequency, correlates, and consequences. Schizophr Bull. 2021;47(5):1261-1287. doi:10.1093/schbul/sbaa181 33459793 PMC8563656

[30] Kessler RC, Berglund P, Demler O, Jin R, Merikangas KR, Walters EE. Lifetime prevalence and age-of-onset distributions of DSM-IV disorders in the National Comorbidity Survey Replication. Arch Gen Psychiatry. 2005;62(6):593-602. doi:10.1001/archpsyc.62.6.593 15939837

[31] Skapinakis P, Lewis G, Davies S, Brugha T, Prince M, Singleton N. Panic disorder and subthreshold panic in the UK general population: epidemiology, comorbidity and functional limitation. Eur Psychiatry. 2011;26(6):354-362. doi:10.1016/j.eurpsy.2010.06.004 20813508

[32] Rim SJ, Hahm BJ, Seong SJ, . Prevalence of mental disorders and associated factors in Korean adults: National Mental Health Survey of Korea 2021. Psychiatry Investig. 2023;20(3):262-272. doi:10.30773/pi.2022.0307 36990670 PMC10064208

[33] Chang HY, Shin YJ, Batty GD, . Measuring depression in South Korea: validity and reliability of a brief questionnaire in the Korean Cancer Prevention Study. J Affect Disord. 2013;150(3):760-765. doi:10.1016/j.jad.2013.02.035 23541487

[34] Kim DM, Bang YR, Kim JH, Park JH. The prevalence of depression, anxiety and associated factors among the general public during COVID-19 pandemic: a cross-sectional study in Korea. J Korean Med Sci. 2021;36(29):e214. doi:10.3346/jkms.2021.36.e214 34313037 PMC8313395

[35] Korean Society for Traumatic Stress Studies. The Fourth National Mental Health Survey: Korean Society for Traumatic Stress Studies. 2021. Accessed May 1, 2024. http://kstss.kr/?p=2065

[36] Lee H, Choi D, Lee JJ. Depression, anxiety, and stress in Korean general population during the COVID-19 pandemic. Epidemiol Health. 2022;44:e2022018. doi:10.4178/epih.e2022018 35057582 PMC9117093

[37] Kim KH, Lee SM, Hong M, Han KM, Paik JW. Changes in mental health service utilization before and during the COVID-19 pandemic: a nationwide database analysis in Korea. Epidemiol Health. 2023;45:e2023022. doi:10.4178/epih.e2023022 36822195 PMC10266929

[38] Chai Y, Man KKC, Luo H, . Incidence of mental health diagnoses during the COVID-19 pandemic: a multinational network study. Epidemiol Psychiatr Sci. 2024;33:e9. doi:10.1017/S2045796024000088 38433286 PMC10940053

[39] Brown WJ. Examining four processes of audience involvement with media personae: transportation, parasocial interaction, identification, and worship. Commun Theory. 2015;25(3):259-283. doi:10.1111/comt.12053

[40] Eaton L. Celebrities help promote campaign to destigmatise mental illness. BMJ. 2009;338:b309. doi:10.1136/bmj.b309 19171557

[41] Thornicroft G, Mehta N, Clement S, . Evidence for effective interventions to reduce mental-health-related stigma and discrimination. Lancet. 2016;387(10023):1123-1132. doi:10.1016/S0140-6736(15)00298-6 26410341

[42] Walsh DAB, Foster JLH. A call to action: a critical review of mental health related anti-stigma campaigns. Front Public Health. 2021;8:569539. doi:10.3389/fpubh.2020.569539 33490010 PMC7820374

[43] Dugan L. The series hazard model: an alternative to time series for event data. J Quant Criminol. 2011;27(3):379-402. doi:10.1007/s10940-010-9127-1

[44] Banerjee A, Dolado JJ, Galbraith JW, Hendry DF. Cointegration, Error Correction, and the Econometric Analysis of Non-Stationary Data. Oxford University Press; 1993.

