# Supplementary material for: Incidence of Panic Disorder Diagnoses After Celebrity Disclosures of Panic Disorder in South Korea
Source: JAMA Netw Open. 2024 Jul 10;7(7):e2420934. doi: 10.1001/jamanetworkopen.2024.20934 (PMC11238026; doi:10.1001/jamanetworkopen.2024.20934)
Supplement: Supplement 1. — eTable 1. Annual Incidence of PD eTable 2. Annual Incidence of OCD eTable 3. Annual Prevalence of PD eTable 4. Annual Prevalence of OCD eFigure 1. Change-Point Analysis of the Incidence of PD eFigure 2. Change-Point Analysis of the Prevalence of PD [file jamanetwopen-e2420934-s001.pdf]

Supplemental Online Content

Kim GE, Jo M, Kim YE, Yoon S, Shin Y. Incidence of panic disorder diagnoses after highly publicized celebrity disclosures of panic disorder in South Korea. *JAMA Netw Open.* 2024;7(7):e2420934. doi:10.1001/jamanetworkopen.2024.20934

eTable 1. Annual Incidence of PD

eTable 2. Annual Incidence of OCD

eTable 3. Annual Prevalence of PD

eTable 4. Annual Prevalence of OCD

eFigure 1. Change-Point Analysis of the Incidence of PD

eFigure 2. Change-Point Analysis of the Prevalence of PD

This supplemental material has been provided by the authors to give readers additional information about their work.

© 2024 Kim GE et al. *JAMA Network Open.*

eTable 1. Annual Incidence of PD (%)

Year	Primary Dx only	~ Secondary Dx	~ Tertiary Dx	~ Quaternary Dx
2004	0.03	0.04	0.05	0.05
2005	0.03	0.05	0.05	0.06
2006	0.04	0.05	0.06	0.06
2007	0.05	0.06	0.07	0.07
2008	0.04	0.06	0.07	0.07
2009	0.04	0.06	0.06	0.07
2010	0.04	0.06	0.07	0.07
2011	0.06	0.14	0.18	0.20
2012	0.08	0.18	0.23	0.26
2013	0.08	0.18	0.23	0.28
2014	0.08	0.19	0.25	0.30
2015	0.10	0.22	0.29	0.34
2016	0.11	0.25	0.33	0.39
2017	0.13	0.28	0.37	0.44
2018	0.15	0.32	0.42	0.50
2019	0.15	0.34	0.45	0.53
2020	0.15	0.35	0.46	0.53
2021	0.18	0.40	0.52	0.61

© 2024 Kim GE et al. *JAMA Network Open.*

eTable 2. Annual Incidence of OCD (%)

Year	Primary Dx only	~ Secondary Dx	~ Tertiary Dx	~ Quaternary Dx
2004	0.01	0.01	0.02	0.02
2005	0.01	0.02	0.02	0.02
2006	0.01	0.02	0.02	0.02
2007	0.02	0.03	0.03	0.04
2008	0.02	0.03	0.03	0.03
2009	0.02	0.03	0.03	0.03
2010	0.02	0.03	0.03	0.04
2011	0.02	0.03	0.03	0.03
2012	0.02	0.03	0.04	0.04
2013	0.02	0.03	0.03	0.04
2014	0.02	0.03	0.03	0.04
2015	0.02	0.03	0.04	0.04
2016	0.02	0.03	0.04	0.05
2017	0.02	0.03	0.04	0.05
2018	0.02	0.04	0.05	0.05
2019	0.02	0.04	0.05	0.06
2020	0.03	0.05	0.06	0.07
2021	0.03	0.06	0.07	0.08

© 2024 Kim GE et al. *JAMA Network Open.*

eTable 3. Annual Prevalence of PD (%)

Year	Primary Dx only	~ Secondary Dx	~ Tertiary Dx	~ Quaternary Dx
2004	0.21	0.33	0.35	0.36
2005	0.26	0.39	0.42	0.43
2006	0.32	0.45	0.48	0.50
2007	0.39	0.53	0.57	0.59
2008	0.44	0.58	0.61	0.64
2009	0.48	0.62	0.66	0.69
2010	0.52	0.67	0.71	0.74
2011	0.60	0.95	1.11	1.20
2012	0.76	1.33	1.65	1.85
2013	0.84	1.57	2.02	2.36
2014	0.90	1.76	2.31	2.75
2015	1.00	2.00	2.67	3.22
2016	1.15	2.31	3.13	3.81
2017	1.31	2.66	3.67	4.51
2018	1.52	3.11	4.31	5.35
2019	1.63	3.46	4.85	6.05
2020	1.76	3.78	5.33	6.67
2021	2.00	4.30	6.03	7.53

© 2024 Kim GE et al. *JAMA Network Open.*

eTable 4. Annual Prevalence of OCD (%)

Year	Primary Dx only	~ Secondary Dx	~ Tertiary Dx	~ Quaternary Dx
2004	0.06	0.10	0.11	0.12
2005	0.07	0.11	0.13	0.13
2006	0.09	0.14	0.15	0.16
2007	0.14	0.21	0.23	0.24
2008	0.16	0.25	0.27	0.28
2009	0.18	0.28	0.30	0.31
2010	0.20	0.30	0.33	0.35
2011	0.21	0.32	0.36	0.37
2012	0.23	0.36	0.40	0.43
2013	0.24	0.37	0.43	0.45
2014	0.25	0.39	0.45	0.48
2015	0.26	0.41	0.48	0.52
2016	0.28	0.44	0.52	0.56
2017	0.29	0.47	0.56	0.61
2018	0.31	0.51	0.61	0.68
2019	0.32	0.55	0.67	0.76
2020	0.35	0.60	0.74	0.85
2021	0.40	0.70	0.87	1.00

© 2024 Kim GE et al. *JAMA Network Open.*

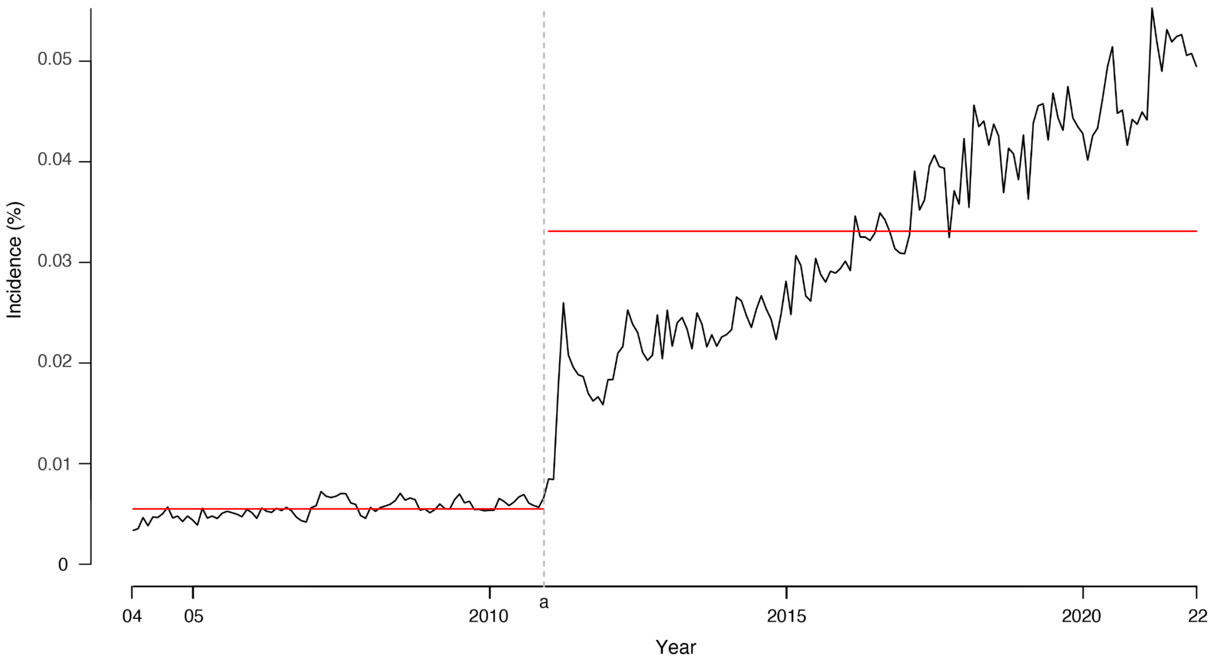

eFigure 1. Change-Point Analysis of the Incidence of Panic Disorder

The solid black line shows the monthly incidence of panic disorder. The vertical dashed black line indicates a) December 2010, when a movie actor made his panic disorder diagnosis public in the media. The red lines show the results of the change-point analysis, indicating that the change occurred in December 2010.

© 2024 Kim GE et al. *JAMA Network Open.*

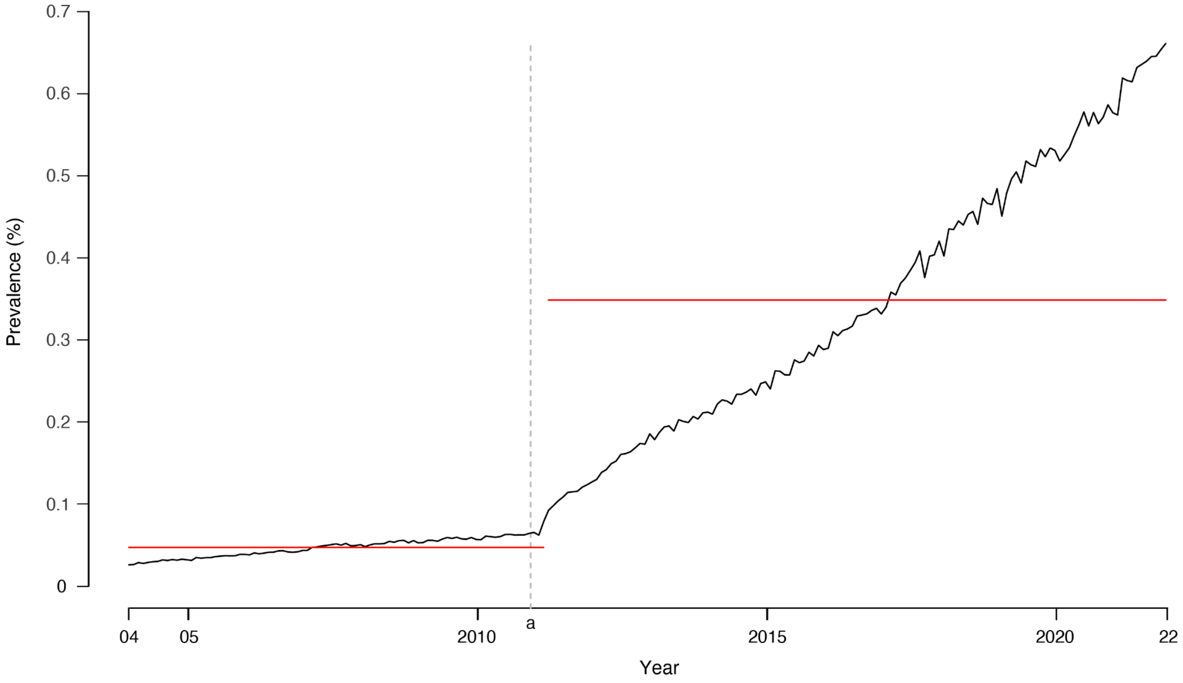

eFigure 2. Change-Point Analysis of the Prevalence of Panic Disorder

The solid black line shows the monthly prevalence of panic disorder. The vertical dashed black line indicates a) December 2010, when a movie actor made his panic disorder diagnosis public in the media. The red lines show the results of the change-point analysis, indicating that the change occurred in March 2011.

© 2024 Kim GE et al. *JAMA Network Open.*
