# Supplementary material for: Incidence of Panic Disorder Diagnoses After Celebrity Disclosures of Panic Disorder in South Korea
Source: JAMA Netw Open. 2024 Jul 10;7(7):e2420934. doi: 10.1001/jamanetworkopen.2024.20934 (PMC11238026; doi:10.1001/jamanetworkopen.2024.20934)
Supplement: Supplement 2. — Data Sharing Statement [file jamanetwopen-e2420934-s002.pdf]

Data Sharing Statement

Data
Data available: Yes
Data types: Deidentified participant data
How to access data: https://nhiss.nhis.or.kr/bd/ab/bdaba000eng.do
When available: With publication

Supporting Documents
Document types: Statistical/analytic code
How to access documents: https://github.com/ad0c1/panicPrevalence
When available: With publication

Additional Information
Who can access the data: Researchers whose proposed use of the data has been approved
Types of analyses: For an academic purpose
Mechanisms of data availability: After approval of a proposal
